# Temporal dynamics and composition of ocular surface microbiota in C57BL/6J mice: uncovering a 12h ultradian rhythm

**DOI:** 10.3389/fcimb.2023.1244454

**Published:** 2023-11-02

**Authors:** Xinwei Jiao, Zhijie Li

**Affiliations:** ^1^ Department of Pathology, Medical School, Jinan University, Guangzhou, China; ^2^ International Ocular Surface Research Center, Institute of Ophthalmology, and Key Laboratory for Regenerative Medicine, Jinan University, Guangzhou, China; ^3^ Department of Ophthalmology, The First Affiliated Hospital of Jinan University, Guangzhou, China

**Keywords:** ocular surface, microbiota, rhythmic oscillations, 16S rRNA, mice

## Abstract

**Purpose:**

This study aimed to investigate the presence of rhythmic fluctuations in the composition, abundance, and functions of commensal core bacteria on the ocular surface of C57BL/6J mice.

**Methods:**

Male C57BL/6J mice, aged 12 weeks, were subjected to a 12-hour light/12-hour dark cycle. Ocular surface tissue samples were collected at four time points (ZT) over a 24-hour period at six-hour intervals. The core ocular surface microbiota’s oscillation cycles and frequencies were assessed using 16S rRNA gene sequencing targeting the V3-V4 region, along with the JTK_CYCLE algorithm. Functional predictions of these bacteria were conducted using PICRUSt2.

**Results:**

Deep sequencing of the ocular surface microbiota highlighted the high abundance of commensal bacteria, with *Proteobacteria*, *Actinobacteriota*, and *Firmicutes* collectively constituting over 90% of the total sample abundance. Among the 22 core bacterial genera, 11 exhibited robust 12-hour rhythms, including *Halomonas*, *Pelagibacterium*, *Pseudomonas*, *Nesterenkonia*, *norank_f_Hyphomonadaceae*, *Stenotrophomonas*, *Anoxybacillus*, *Acinetobacter*, *Zoogloea*, *Brevibacillus*, and *Ralstonia*. Further taxonomic analysis indicated significant intra-cluster similarities and inter-cluster differences at the order, family, and genus levels during ZT0/12 and ZT6/18. Community interaction networks and functional prediction analyses revealed synchronized 12-hour rhythmic oscillations in neural, immune, metabolic, and other pathways associated with symbiotic bacteria.

**Conclusion:**

This study demonstrates the presence of ultradian rhythmic oscillations in commensal bacteria on the ocular surface of normal C57BL/6J mice, with a 12-hour cycle. These findings suggest a crucial role for ultradian rhythms in maintaining ocular surface homeostasis in the host.

## 1 Introduction

The human body harbors a vast and complex population of microorganisms that exceeds the total number of human cells by several orders of magnitude (Sender et al., 2016; Mukherjee et al., 2023). These microorganisms primarily inhabit the interfaces between the body and the external environment, including areas such as the skin, gut, mouth, and vagina (Murray et al., 2007; Silva et al., 2015). They engage in complex and diverse interactions with the host, a role that is of paramount importance in maintaining physiological homeostasis and serving as a protective barrier against the onset and progression of various diseases (Yang et al., 2018; Lahiri et al., 2019; Sun et al., 2022). Perturbations in these commensal microorganisms can lead to a spectrum of pathological changes and even contribute to the development of specific diseases (Liguori et al., 2016; Hu et al., 2022; Liu et al., 2022; Zhao et al., 2022). Consequently, a comprehensive understanding of the patterns of variation within these commensal microorganisms at various body interfaces under both normal physiological and pathological conditions is a central goal of contemporary biomedical research.

Of particular interest is the ocular surface, an exceptional body interface comparable to mucous membranes, which harbors a diverse microbiota despite the continuous rinsing action of the tear film and the presence of a number of antimicrobial molecules (Human Microbiome Project, 2012; McDermott, 2013; Gevers et al., 2014). This ocular microbiota has been shown to exert a competitive inhibitory influence, effectively preventing the colonization of pathogenic bacteria (Aragona et al., 2021). Nevertheless, the diversity and abundance of this microbiota on the human ocular surface can exhibit significant variability due to a myriad of factors, including geographic location, dietary habits, age, gender, use of ocular prostheses, and the presence of systemic or localized diseases (Wen et al., 2017; Chao et al., 2018; Deng et al., 2020; Gomes et al., 2020; Li et al., 2021; Qi et al., 2021). In It is worth noting that despite the widespread use of the C57BL/6J mouse model in basic ophthalmic research, in-depth investigations of the mouse ocular surface microbiome at different taxonomic levels remain relatively scarce.

Recent research efforts have revealed distinct characteristics and temporal fluctuations in the microbiota inhabiting different body interfaces. For example, the gut microbiota of a normal mouse exhibits a circadian rhythm that adheres to a 24-hour cycle, a rhythm that can be disrupted in mice with genetic deficiencies in core clock genes that control circadian rhythms (Thaiss et al., 2016). These findings underscore the complex interplay between the host and its commensal microbiota. In addition, previous studies have demonstrated significant circadian rhythmicity in critical ocular components, such as the lacrimal gland, which is responsible for tear film secretion, and the cornea, which is located at the front of the eye (Jiao et al., 2019; Jiao et al., 2020; He et al., 2021; Jiao et al., 2021). However, whether the microbiota populating the ocular surface exhibit analogous rhythmic oscillations remains an unresolved question that warrants further investigation.

In light of the above considerations, we hypothesized that the diverse core commensal bacteria inhabiting the ocular surface of C57 mice may exhibit distinct rhythmic oscillations over time. To rigorously investigate this hypothesis, we meticulously collected ocular surface tissues at six-hour intervals over the course of a complete 24-hour circadian cycle. We then performed a comprehensive and longitudinal analysis using deep sequencing of the 16S rRNA gene. Our results support the presence of an ultradian rhythmic oscillation within the ocular surface flora. This rhythmic pattern manifests with a precise 12-hour cycle, suggesting a complex temporal regulation of these microbial communities. Furthermore, our functional predictions, which are intimately linked to this oscillatory flora, consistently demonstrate 12-hour ultradian rhythmic oscillations. These empirically based results not only elucidate the fundamental nature of ocular surface microbes, but also shed light on their potential implications in the context of disease pathology. Finally, our observation may open new avenues for the development of innovative therapeutic interventions in the field of ocular health and underscores the importance of temporal dynamics within microbial communities in ocular ecosystems.

## 2 Materials and methods

### 2.1 Overall study design

**Figure 1:**
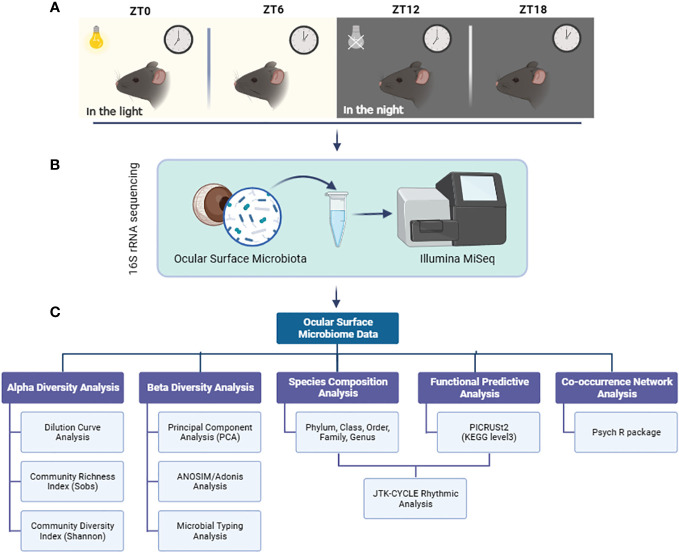
Experimental design and workflow. **(A)** Ocular surface tissues from mice were collected at 4 time points at 6-hour intervals during a light cycle; **(B)** 16S rRNA sequencing of microbiota in ocular surface tissues using the Illumina MiSeq platform; **(C)** Multiple bioinformatics analysis and JTK CYCLE rhythmicity identification were performed on the sequencing data.

Our study included a well-defined experimental design and workflow: Ocular surface tissues, including the entire conjunctiva and cornea, were collected from six male C57bl/6J mice at four different times during a 24-hour diurnal cycle: ZT0, ZT6, ZT12, and ZT18 (**Figure 1A**). We sequenced the microbial flora in these ocular surface tissues using 16S rRNA gene sequencing on the Illumina MiSeq 2 x 300 bp platform (**Figure 1B**). The sequencing data were then thoroughly analyzed using bioinformatics techniques, including diversity analysis, species composition assessment, PICRUSt2 functional prediction, and co-occurrence network analysis. In addition, we used the JTK-CYCLE algorithm to perform rhythmic analyses of the dominant bacterial genera and their predicted functional enrichment within the KEGG pathway in the mouse ocular surface at the four designated time points (**Figure 1C**).

### 2.2 Experimental animals

Male C57BL/6J mice, aged 12 weeks, were procured from Guangdong GemPharmatech Co., Ltd (Guangzhou, China). These mice adhered to a strict 12-hour light/12-hour dark cycle, receiving care under optimal temperature and humidity conditions. This study adopted the Zeitgeber time scale for timing, with ZT0 indicating lights on at 7:00 AM and ZT12 representing lights off at 7:00 PM. The mice had access to food and water *ad libitum*. All experimental procedures and animal care protocols strictly adhered to the guidelines outlined in the Association for Research in Vision and Ophthalmology (ARVO) Statement for the Use of Animals in Ophthalmic and Vision Research. Our study received approval from the Animal Care and Use Committee of Jinan University. At the conclusion of the study, humane euthanasia was administered to the mice through inhalation of an isoflurane overdose, followed by cervical dislocation.

### 2.3 Sampling of the ocular surface microbiota

To ensure complete collection of ocular surface tissue, we commenced the procedure by making incisions in the skin on the lateral side of both eyelid margins. This maneuver exposed all of the ocular surface tissue as we gently pressed the eyelids around the eye. Intact conjunctival and corneal tissue was then removed with sterile scissors, carefully separating the conjunctiva from the eyelids and the connective tissue around the eyeball. These samples were rapidly frozen in liquid nitrogen and stored at -80°C. To ensure the absence of bacterial contamination during sampling, frozen tubes without samples were included as negative controls. In addition, eyelid skin samples were collected as positive controls to exclude contamination of the ocular surface flora from the surrounding eyelids. Every aspect of the procedure was performed in a sterile environment within an ultra-clean bench to avoid any potential contamination.

### 2.4 16S rRNA gene sequencing

For the extraction of genomic DNA from ocular surface bacteria in each sample, we employed a DNA extraction kit (Omega Bio-tek, Norcross, GA, USA), following the manufacturer’s guidelines. Integrity of the extracted genomic DNA was verified through 1.2% agarose gel electrophoresis. We targeted the V3-V4 region of 16S rDNA and amplified the desired fragment using universal primers 338F (5 ‘- ACTCCTACGGGAGGCAGCAG-3’) and 806R (5 ‘- GGACTACHVGGGTWTCTAAT-3’). Library construction involved a two-step PCR amplification process. PCR products were subsequently recovered using the AxyPrepDNA gel recovery kit (AXYGEN Corporation) and quantified via fluorescence measurements on an FTC-3000TM Real-Time PCR instrument. Following homogenization and mixing, library construction was completed, and sequencing was carried out on an Illumina MiSeq 2 x 300 bp platform.

After sequencing, we analyzed the obtained reads using QIIME2 software (http://qiime2.org/). Chimeric sequences were identified and eliminated using the USEARCH algorithm, and an open reference operational taxonomic unit (OTU) approach was adopted. The Greengenes 16S rRNA gene database (version 13-5, 97%) was utilized for classification, resulting in the organization of data into a BIOM format OTU table in QIIME2. Quality control for the original sequencing sequences was conducted using Trimmomatic software, and FLASH software was employed for assembly. To cluster OTU sequences at a 97% similarity threshold, UCHIME software was employed to eliminate chimeras. The assignment of each sequence to a specific species was performed using the Silva database (silva138/16s_bacteria) and the RDP classifier (http://rdp.cme.msu.edu/) with an alignment threshold of 70%.

### 2.5 Bioinformatics analysis of ocular surface microbiome

Alpha In our quest to delve into the intricacies of the ocular surface microbiome, we employed a comprehensive array of bioinformatics tools and methodologies. To assess the alpha diversity of the microbiome at the OTU level, we utilized Mothur software. Our analysis encompassed several essential indices, including those for community abundance (Sobs) and community diversity (Shannon). Additionally, we employed rarefaction curve analysis to ascertain whether our sequencing depth adequately captured the microbial diversity in all samples.

To discern any significant differences between index groups, we performed Student’s t-tests. Beta diversity among the samples was evaluated using the Fast Unifrac method, and we harnessed principal component analysis (PCA) for visualization. To delve deeper into the dominant flora structure across different samples, we calculated Jensen-Shannon Distances (JSD) based on their relative abundances at various taxonomic levels. For microbiota typing analysis, we employed PAM (Partitioning Around Medoids) clustering.

Taxonomy was meticulously assigned using Silva as the reference database. We meticulously examined the community composition of each sample across multiple taxonomic levels, ranging from phylum to genus. For a deeper understanding of microbial functionality, we employed PICRUSt2 to predict functional profiles based on 16S rRNA gene sequences, which were represented as Kyoto Encyclopedia of Genes and Genomes (KEGG) Orthology (KO).

To unravel the intricate microbial interactions on the ocular surface at different time points, we performed bacterial genomic binning assembly based on 16S rRNA sequencing data. Subsequently, we conducted network co-occurrence analysis, leveraging the bin information from the Psych R package, followed by visualization using GEPHI. Our analysis included the calculation of Spearman correlations, with a focus on retaining statistically significant correlations (p < 0.05) for further exploration.

### 2.6 Rhythmic analysis of ocular surface community composition and function

To probe the rhythmic patterns within the ocular surface microbiome, we harnessed the JTK_CYCLE algorithm, implemented using the R language (Hughes et al., 2010). Our objective was to identify cycling variables within the vast dataset. For this analysis, we inputted abundance values of bacterial genera over time, with a specific focus on identifying significant oscillatory cycles and the specific bacteria associated with them (Benjamini–Hochberg Q values (BH.Q) < 0.05).

In parallel, we subjected the KEGG pathways predicted from microbial genes using PICRUSt2 to the JTK_CYCLE algorithm. This allowed us to identify functional pathways exhibiting significant oscillations (BH. Q < 0.01). Given our sampling intervals of 6 hours throughout the day, totaling four time points and with six biological replicates at each time point, our JTK_CYCLE code configuration was as follows: (jtkdist(4, 6), periods <- 2:4, jtk.init(periods, 6)). All other parameters were maintained at their default settings.

Through this comprehensive analysis, we also determined the phase and amplitude of rhythmic bacteria and pathways, providing a deeper understanding of the temporal dynamics within the ocular surface microbiome.

## 3 Results

### 3.1 The alpha diversity of the ocular surface microbiota has a dynamic fluctuation

**Figure 2:**
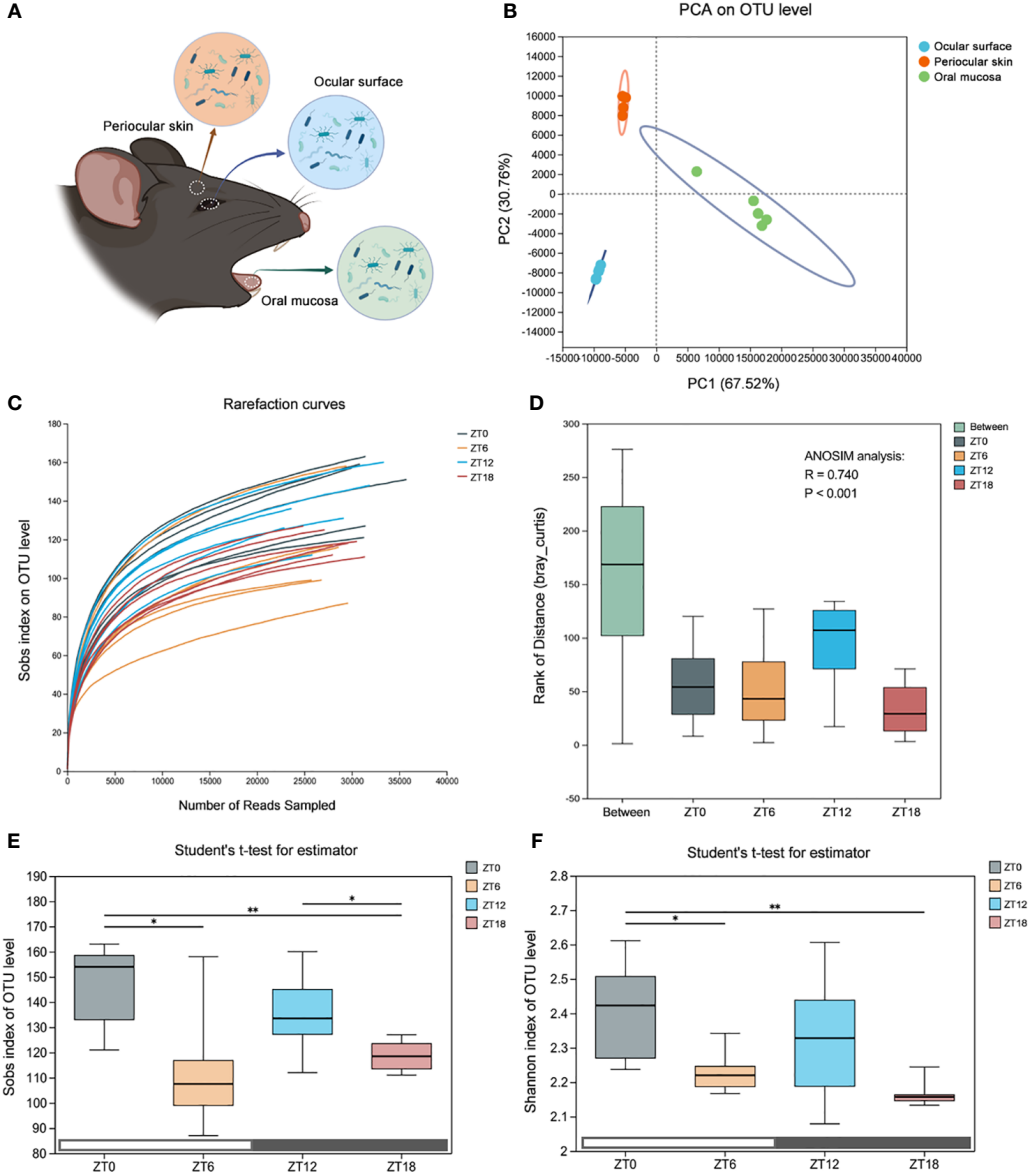
Comparison of the ocular surface microbiota alpha diversity at four time points. **(A)** Schematic diagram of the microbiota sampling from the mouse ocular surface, periocular skin and oral mucosa. **(B)** Principal component analysis (PCA) score plots based on the OTU abundance of the three sites. **(C)** Refraction curves of the Sobs index based on the OTU level. **(D)** ANOSIM similarity analysis. The box “Between” refers to the differences between groups, and the others show the differences within their respective groups. R values represent the explanation of sample differences by grouping factors, and P values represent statistical differences. **(E, F)** Sobs index and Shannon index based on OTU level, which represent community abundance and diversity, respectively. Data are presented as mean ± SD, Student′s t-test, *P<0.05, **P < 0.01.

In our pursuit of understanding the dynamics of alpha diversity in the ocular surface microbiota, we implemented a comprehensive approach. Initially, to rule out any potential bacterial contamination during sampling, we concurrently collected samples from the ocular surface, periocular skin, and oral mucosa. These samples were then subjected to 16S rRNA sequencing to discern any biogeographic distribution disparities among these locations (**Figure 2A**). Subsequently, we employed PCA analysis at the OTU level to confirm the distinctiveness of microbiota at these three sites (**Figure 2B**). With this foundational understanding, we proceeded to construct rarefaction curves based on the Sobs index at the OTU level. Our aim was to explore how ocular surface species’ abundance varied with different sequencing depths (**Figure 2C**). The insightful outcome revealed that, as sequencing depth increased, all samples exhibited a gently ascending curve. This observation indicated that the volume of sequencing data we amassed was sufficient to encapsulate the diversity of the majority of microorganisms on the mouse ocular tissue surface, even within the cryptic conjunctival folds.

To delve deeper into the dynamic nature of the ocular surface commensal flora, we established a sampling regimen with 6-hour intervals over a 24-hour light/dark cycle (ZT0, ZT6, ZT12, and ZT18). Subsequently, we conducted comprehensive deep 16S rRNA sequencing. Our data underwent rigorous statistical scrutiny through the non-parametric ANOSIM test, revealing a significant statistical difference (R=0.740, P<0.001) among the time points (**Figure 2D**).

To assess alterations in the alpha diversity of the ocular surface symbiotic flora at each of the four time points, we leveraged the OTU-based Sobs index (indicating community abundance) and the Shannon index (indicating community diversity) (**Figures 2E, F**). Our findings unveiled that the abundance and diversity of the ZT0/12 flora generally exceeded those of ZT6/18. Importantly, these index values exhibited a distinct tendency to fluctuate over time. Consequently, these data affirm that the diversity and abundance of the ocular surface flora undergo dynamic fluctuations within the span of a single day. Moreover, the longitudinal time stamps underscore that alpha-diversity follows a dynamic pattern, characterized by two oscillatory cycles within a light-dark cycle, initially peaking and subsequently declining.

### 3.2 Composition of ocular surface communities at different taxonomic levels

**Figure 3:**
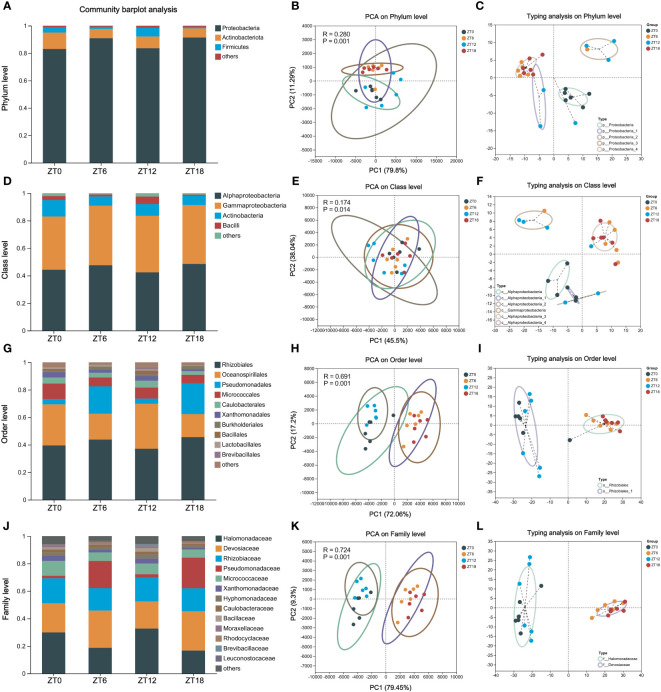
Composition of the ocular surface core flora at different taxonomic levels. Phylum **(A-C)**, class **(D-F)**, order **(G-I)** and family **(J-L)** -level community composition analysis, PCA (intergroup difference test: ANOSIM) and microbiota typing analysis on OS. In all samples, species with less than 1% abundance were classified as “others”.

In our pursuit to uncover the core microbiota inhabiting the ocular surface of C57BL/6J mice, we embarked on an in-depth analysis of the 16S rRNA sequencing data. Employing QIIME (version 1.9.1) and RDP Classifier (version 2.13) software, we scrutinized the data at various taxonomic levels, including phylum, class, order, family, and genus. The initial findings unveiled a prominent presence of three core bacterial phyla: *Proteobacteria*, *Actinobacteriota*, and *Firmicutes*. Impressively, these phyla collectively accounted for more than 90% of the microbial composition across all sampling time points (**Figure 3A**). Intriguingly, there was no difference in phylum-level similarity of bacteria among the four sampling times, as shown by PCA (**Figure 3B**).

Further delving into the bacterial community, we observed that a significant proportion of samples could be classified under the *Proteobacteria* category, consistently exceeding 80% relative abundance across all four time points (**Figure 3C**). Zooming in at the class level, *Alphaproteobacteria*, *Gammaproteobacteria*, *Actinobacteria*, and *Bacilli* stood out as the dominant players (**Figure 3D**). Notably, PCA analysis continued to underscore the lack of significant differences in the temporal progression of these samples (R=0.169, P=0.016) (**Figure 3E**). The *AlphaProteobacteria* and *GammaProteobacteria* were the prevalent bacterial types across these samples, each contributing 40% (**Figure 3F**).

Descending further into the hierarchy to the bacterial order level, we identified ten core bacterial species, collectively accounting for up to 80% of the flora’s abundance in our samples (**Figure 3G**). Interestingly, PCA clustering indicated a distance difference between ZT0/12 and ZT6/18 (R=0.690, P=0.001), signaling variations in bacterial counts throughout the day (**Figure 3H**). Subtyping analysis validated this observation, with *Rhizobiales* emerging as the common subtype between the two clusters (**Figure 3I**). Finally, our exploration extended to the bacterial family level, where we identified a total of 13 species within the core microbiota (**Figure 3J**). Here, once again, ZT0/12 and ZT6/18 clusters exhibited pronounced intra-cluster similarity and inter-cluster differences (R=0.724, P=0.001) (**Figure 3K**). Notably, these clusters were characterized by an abundance of *Halomonadaceae* and *Devosiaceae*, respectively (**Figure 3L**).

In summary, our comprehensive analysis has illuminated the presence of a substantial core microbiota at multiple taxonomic levels on the ocular surface of C57BL/6J mice. Furthermore, we’ve observed that the microbiota at the order and family levels exhibit temporal longitudinal differences, adding depth to our understanding of this fascinating ecosystem.

### 3.3 The ocular surface bacterial genus exhibits 12h ultradian rhythms

**Figure 4:**
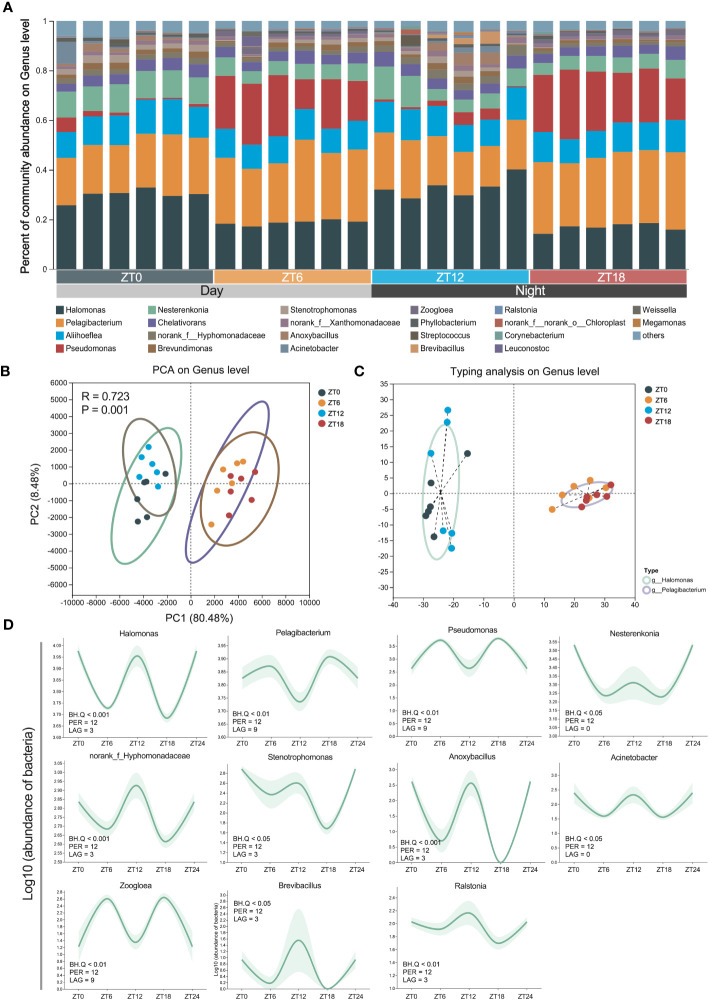
Rhythmic oscillations of ocular surface genus-level bacteria. **(A)** The composition analysis of ocular surface core microbiota community. Species with less than 1% abundance in all samples were classified as “others”. **(B)** PCA analysis of mouse eye samples at 4 sampling time points. **(C)** ocular surface microflora typing analysis of bacterial genera. **(D)** Confidence line plot of 12-h rhythmic ocular surface bacterial genera based on Figure **(A)** Confidence: 0.9, JTK_CYCLE algorithm screening threshold: *BH.Q* < 0.05.

In our quest to explore time-dependent variations in ocular surface commensal bacteria at the genus level, we delved into the core bacterial flora residing on the mouse ocular surface. Our investigation uncovered a roster of 22 core bacterial genera, each with a relative abundance exceeding 1%. Notably, the top five genera in terms of abundance included *Halomonas*, *Pelagibacterium*, *Aliihoeflea*, *Pseudomonas*, and *Nesterenkonia* (**Figure 4A**). What’s particularly intriguing is that our PCA analysis revealed distinct sample clustering at ZT0/12 and ZT6/18, aligning with our earlier findings at the order and family levels (**Figure 4B**). Among these clusters, *Halomonas* and *Pelagibacterium* emerged as the most predominant, collectively constituting 30% of the samples in each bacterial cluster (**Figure 4C**). To unravel the oscillatory patterns within these clusters over time, we employed the JTK_CYCLE algorithm to scrutinize all genus-level microbiota in the ocular surface samples collected at four distinct time points. Our analyses unveiled a compelling result: approximately 50% of the microbial clusters exhibited significant 12-hour rhythms (BH. Q < 0.05). In precise terms, 11 out of the 22-core ocular surface bacterial genera demonstrated remarkable 12-hour rhythms. These included *Halomonas*, *Pelagibacterium*, *Pseudomonas*, *Nesterenkonia*, *norank_Hyphomonadaceae*, *Stenotrophomonas*, *Anoxybacillus*, *Acinetobacter*, *Zoogloea*, *Brevibacillus*, and *Ralstonia* (**Figure 4D**). In summary, our comprehensive analysis has unveiled the presence of a 12-hour rhythmicity in bacterial clustering at the genus level. It’s noteworthy that ZT0/ZT12 and ZT6/ZT18 represent pivotal time points marking the onset of these rhythmic variations.

### 3.4 The ocular surface microbial co-occurrence network and predicted functions have a 12-h ultradian rhythm

**Figure 5:**
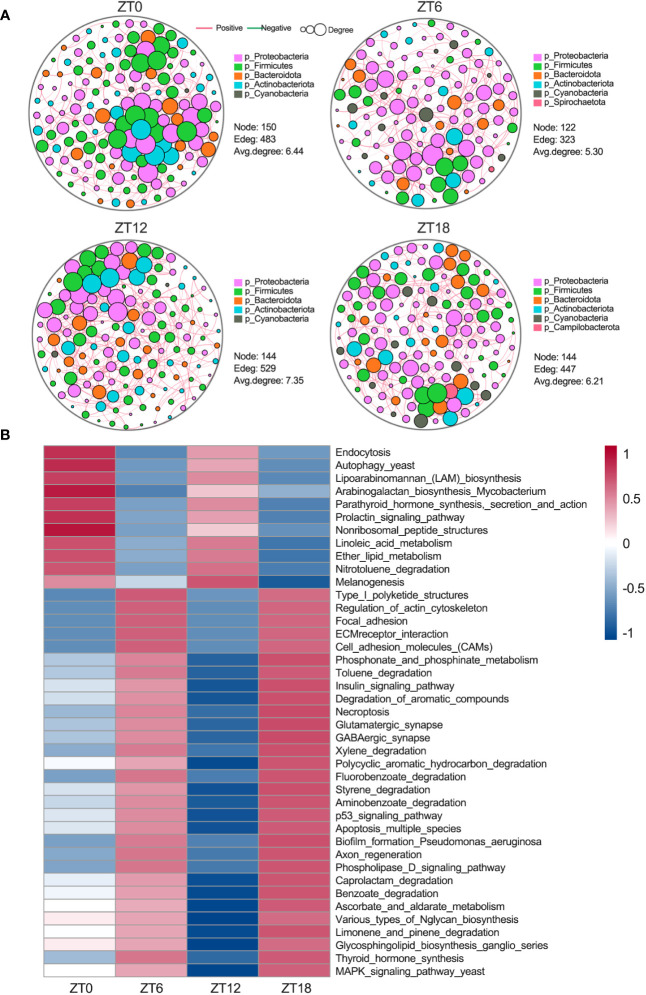
Ocular surface microbial interactions and rhythmic analysis of the KEGG pathway. **(A)** Microbial co-occurrence network analysis at the phylum level. Red lines show positive correlations and green lines indicate negative correlations. Each node represents a microorganism, and each edge indicates the interaction relationship between microorganisms. The area of the node represents the degree, and the degree is the number of nodes directly connected to this node. **(B)** PICRUSt2 was used to predict ocular surface microbial function at the third level of the KEGG pathway, followed by the JTK_CYCLE algorithm for rhythmic screening (*BH.Q* < 0.01).

In our pursuit of a deeper understanding of the ocular surface microbiota’s dynamics, we conducted an analysis of bacterial population co-occurrence network patterns within the sample communities, leveraging the Psych R package. The outcomes unveiled distinct interaction patterns of bacterial networks at different times of the day (**Figure 5A**). Notably, these patterns’ complexity primarily hinged on the average degree. Specifically, we observed fluctuations following a high-low-high-low pattern. These fluctuations indicate that interactions within the ocular surface microbiota undergo two distinct oscillations within a single light-dark cycle. Taken together, these findings strongly suggest that the ocular surface microbiota undergoes dynamic changes throughout the day, which may hold significant implications for the maintenance of ocular surface health.

To shed light on the biological significance of the temporal co-occurrence network patterns observed within the ocular surface microbiota, our first step was to employ the PICRUSt2 software for predicting functional KEGG enrichment of bacterial genes at four different ZT (zeitgeber time) points. Subsequently, we applied the JTK_CYCLE algorithm (*BH.Q* < 0.01) to discern the rhythmicity of these pathways. Notably, our findings revealed that 41 KEGG pathways displayed significant enrichment and exhibited an oscillatory pattern consistent with the 12-hour cycle observed in the co-occurrence network (**Figure 5B**). Among these 41 enriched pathways, 11 showed activations at ZT0 and ZT12, primarily associated with sugar synthesis and fatty acid metabolism. In contrast, the remaining 30 pathways displayed significant activation at ZT6 and ZT18. These pathways were mainly associated with three key categories: (1) cell motility and adhesion, encompassing regulation of actin cytoskeleton, focal adhesion, ECM receptor interactions, and cell adhesion molecules (CAMs); (2) neural activity, including glutamatergic synapses, GABAergic synapses, and axonal regeneration; and (3) cell death, spanning the p53 pathway, the yeast MAPK pathway, necroptosis, and apoptosis across multiple species. Collectively, these results underscore the 12-hour cycle characterizing the co-occurrence network of the ocular surface microbiota and its underlying biological functions, emphasizing the pivotal role of temporal dynamics in shaping the microbial community and its functional contributions to the host.

## 4 Discussion

This study examines the diurnal variation of commensal bacteria on the ocular surface of male C57BL/6J mice housed in a pathogen-free environment using 16S rRNA sequencing. The results reveal a remarkable discovery: nearly 50% of the commensal bacterial genera on the ocular surface exhibit ultradian rhythms with a 12-hour period, corresponding to the typical 12-hour light/12-hour dark cycle. Furthermore, the functional pathways and community interaction networks involving ocular surface commensal bacteria and their metabolites, spanning neural, immune, and metabolic systems, also exhibit a 12-hour oscillatory pattern. Thus, this study highlights the importance of considering the ultradian rhythm of ocular surface commensal bacteria when investigating their role in ocular surface health and related diseases.

Recent studies have highlighted the critical role of the ocular surface microbiome in maintaining ocular health and protecting against pathogenic colonization. Alterations in this microbial ecosystem have been linked to several ocular and systemic diseases (St Leger and Caspi, 2018; Petrillo et al., 2020). Our research has shown that the composition of the ocular surface microbiota in C57BL/6J mice is similar to that of healthy humans, with the phyla *Proteobacteria*, *Actinobacteriota*, and *Firmicutes* dominating. In addition, core microbiota including *Bacillus*, *Acinetobacter*, *Stenotrophomonas*, *Streptococcus*, and *Pseudomonas* have been identified (Dong et al., 2011; Zhou et al., 2014; Huang et al., 2016; Ozkan et al., 2017; Ozkan et al., 2021; Lee et al., 2022). These findings suggest the suitability of the C57BL/6J mouse model for investigating the relationship between ocular disease and changes in the ocular surface microbiota.

While most biological activities follow a 24-hour circadian rhythm driven by the Earth’s rotation and solar illumination, recent studies have shown that the composition and abundance of the microbiota in the gut, skin, and oral cavity of rodents also exhibit circadian rhythms (Wilkins et al., 2021; Chellappa et al., 2022). Interestingly, our preliminary data indicate that the ocular surface microbiota of male C57BL/J mice exhibit an unusual 12-hour ultradian rhythmic oscillation. The causes and mechanisms behind this phenomenon remain unclear. One hypothesis links it to the origin of life in the ocean, where marine animals synchronized their behavior with the 12-hour tidal cycle due to the gravitational pull of the moon (Satoh et al., 2008; Wilcockson and Zhang, 2008; Last et al., 2009; Zhang et al., 2013; Tran et al., 2020). Similarly, certain physiological activities in mammals have evolved to maintain a 12-hour ultradian rhythm, as evidenced by transcripts with 12-hour periodicity in various tissues (Hughes et al., 2009). Our previous study even detected a 12-hour transcriptional rhythm in mouse corneal tissue, which is critical for coordinating cellular stress and systemic homeostasis (Jiao et al., 2019). Dysregulation of this rhythm increases the risk of metabolic disease (Zhu et al., 2017; Meng et al., 2022; Asher and Zhu, 2023). While more research is needed, it’s clear that microbiota in different body regions exhibit time-dependent oscillations with potential implications for host health.

Maintaining ocular surface homeostasis is critical to preventing infection and preserving visual function. The balance of host-microbiota interactions involves multiple functions, including barrier protection, inhibition of apoptosis and inflammation, rapid wound healing, rejection of potential pathogens, and maintenance of immune tolerance (Kugadas et al., 2017; Gomes et al., 2020; Kang et al., 2020; Petrillo et al., 2020). Our functional prediction analysis highlights the dynamic nature of ocular surface microbiota composition and function following a 12-hour cycle. This dynamic property allows the microbiota to adapt to environmental changes and anticipate fluctuating physiological demands of the host (Moye et al., 2014; Jehrke et al., 2018; Gomez-Alvarez et al., 2021). Notably, pathways involved in glycan synthesis and fatty acid metabolism show inverse timing with the activation of cytoskeletal, cell adhesion, neural, and immune pathways. This suggests that periodic niche occupancy by specialists responding to the periodic physiological demands of the host ocular tissues may lead to these functional fluctuations. Non-oscillating species may play a “housekeeping” role unaffected by diurnal variation. Thus, coordination between periodic and aperiodic microbiota functions likely contributes to the maintenance of functional homeostasis on the host ocular surface.

Several considerations should be taken into account when interpreting the data from this study. First, ocular surface microbiome samples should be collected in conjunction with samples from the lid margin and periocular skin to eliminate the possibility of contamination by commensal microorganisms on the periocular tissue surface. Second, while 16S rRNA sequencing accurately identifies and classifies genetic fragments of the mouse ocular surface microbiota, it doesn’t distinguish between living and dead bacteria. Third, the interaction between the 12-hour ultradian rhythm of the ocular surface microbiota and the 24-hour circadian rhythmicity of host ocular surface tissues, particularly the cornea, requires further investigation. Finally, additional research is needed to elucidate the mechanisms that contribute to and maintain the ultradian rhythm of the ocular surface microbiome and its role in ocular surface health and disease.

In conclusion, this study provides new insights into the ultradian oscillation of species composition and predicted biological functions within the ocular surface microbiota of C57BL/6J mice under healthy conditions. It highlights the potential importance of the physiological rhythmicity of the microbiota in maintaining ocular surface homeostasis. It also highlights the importance of timing ocular surface sampling in future research efforts.

## Data availability statement

The data presented in the study are deposited in the NCBI SRA repository, accession number: PRJNA987049; PRJNA1018812. https://www.ncbi.nlm.nih.gov/bioproject/?term=PRJNA987049; https://www.ncbi.nlm.nih.gov/bioproject/?term=PRJNA1018812.

## Ethics statement

The animal study was approved by Animal Care and Use Committee of Jinan University. The study was conducted in accordance with the local legislation and institutional requirements.

## Author contributions

ZL conceived and designed experiments, drafted the manuscript, and approved the final version. XJ performed the experiments, analyzed and interpreted the data. XJ contributed reagents/materials/analysis tools. All authors have approved the final version of the manuscript.
